# Niche partitioning in an assemblage of granivorous rodents, and the challenge of community-level conservation

**DOI:** 10.1007/s00442-021-05104-5

**Published:** 2022-01-15

**Authors:** Rachel Y. Chock, Debra M. Shier, Gregory F. Grether

**Affiliations:** 1 Department of Ecology and Evolutionary Biology, University of California, Los Angeles, CA USA; 2 Present Address: Recovery Ecology, San Diego Zoo Wildlife Alliance, Escondido, CA USA

**Keywords:** Coexistence, Resource selection, Competition, Pocket mouse, Kangaroo rat

## Abstract

Coexistence of competing species in the same foraging guild has long puzzled ecologists. In particular, how do small subordinate species persist with larger dominant competitors? This question becomes particularly important when conservation interventions, such as reintroduction or translocation, become necessary for the smaller species. Exclusion of dominant competitors might be necessary to establish populations of some endangered species. Ultimately, however, the goal should be to conserve whole communities. Determining how subordinate species escape competitive exclusion in intact communities could inform conservation decisions by clarifying the ecological conditions and processes required for coexistence at local or regional scales. We tested for spatial and temporal partitioning among six species of native, granivorous rodents using null models, and characterized the microhabitat of each species using resource-selection models. We found that the species’ nightly activity patterns are aggregated temporally but segregated spatially. As expected, we found clear evidence that the larger-bodied kangaroo rats drive spatial partitioning, but we also found species-specific microhabitat associations, which suggests that habitat heterogeneity is part of what enables these species to coexist. Restoration of natural disturbance regimes that create habitat heterogeneity, and selection of translocation sites without specific competitors, are among the management recommendations to consider in this case. More generally, this study highlights the need for a community-level approach to conservation and the usefulness of basic ecological data for guiding management decisions.

**Supplementary Information:**

The online version contains supplementary material available at 10.1007/s00442-021-05104-5.

## Introduction

It is still an open question how multiple species that are similar in diet and habitat use can persist together in communities (Hutchinson 1961; Robinson and Terborgh 1995; Manlick et al. 2021). Community assembly rules and coexistence theory can provide a basis for investigating co-occurrence (Diamond 1975; Chesson 2000; HilleRisLambers et al. 2012). While environmental conditions drive large-scale biogeographic patterns in species diversity (Wiens and Donoghue 2004), competitive interactions within neighborhoods are important contributors to local coexistence (Chesson 2000). If a superior competitor causes a decline in the population growth rate of an inferior competitor, the result may be competitive exclusion—the local extirpation of the inferior competitor. Interactions between species may be in the form of interference competition, where interspecific aggression occurs over shared resources, or exploitative competition, where species interact indirectly by depleting a shared, limiting resource. Interference and exploitative competition are generally expected to reduce the probability of species coexistence, however, under some circumstances interspecific competition can stabilize coexistence (Amarasekare 2002; Grether et al. 2017).

Community assembly and coexistence theory (Chesson 2000; HilleRisLambers et al. 2012) are seldom applied to conservation but might be more relevant than is generally recognized. Once a species has been lost from a community, or has declined to the point of being functionally absent, competition with other native species can be an impediment to recovery (Hamel et al. 2013; Christensen et al. 2019; Berger-Tal et al. 2020). Conservation interventions that give at-risk species a short-term advantage by suppressing competitors are not likely to be sustainable or desirable in the long term. Determining how such species coexist with competitors in intact communities could lead to more effective conservation management.

Various mechanisms have been proposed to stabilize coexistence in assemblages of granivorous rodents, but most proposed mechanisms can be characterized as a type of spatial or temporal niche partitioning that reduces interspecific competition relative to intraspecific competition (Schoener 1974; Price 1978; Brown 1989). Spatial and temporal separation between rodent species often arises from interference competition. Larger-bodied species typically dominate smaller species in direct interactions, and it is common for the activity patterns of subordinate species to shift away from those of dominant competitors (Glass and Slade 1980; Ziv et al. 1993; Gutman and Dayan 2005; Pasch et al. 2013). As examples: (1) two species of desert gerbils (*Gerbillus* spp.) prefer the same habitat type in allopatry, but in sympatry the subordinate species mainly uses a secondary habitat type and forages later at night than the dominant species (Ziv et al. 1993); (2) golden spiny mice (*Acomys russatus)* are normally active only during the day, but when a dominant and nocturnal congener was experimentally excluded, they became active at night as well (Gutman and Dayan 2005); and (3) the spatial and diel activity patterns of prairie voles (*Microtus ochrogaster*) shift seasonally as a direct consequence of avoiding encounters with a larger-bodied competitor (Glass and Slade 1980). Such shifts in activity patterns can result in subordinate species foraging at times or locations where predation risk is higher, or where resources are less abundant, with negative effects on individual fitness and the size of the population (Glass and Slade 1980). Nevertheless, the resulting niche partitioning might be what enables the species to coexist (Carothers and Jaksić 1984; Ziv et al. 1993).

Heteromyidae, a family of rodents that includes kangaroo rats, pocket mice and kangaroo mice, often live in multispecies assemblages forming a seed-foraging guild (Brown and Harney 1993). Through seed predation and soil disturbance, heteromyids can function as keystone species (Brown and Heske 1990; Goldingay et al. 1997; Davidson and Lightfoot 2006). Interspecific competition has been documented by experimentally removing one species and quantifying the effect on other species in the community. Brown and Munger (1985) documented delayed increases in densities of rodent species in the same seed foraging guild as the removed species, but no increase of insectivorous rodents, consistent with the idea that interspecific exploitative competition for food regulates population densities. In other experiments, the removal of large-bodied species had positive effects on the density of small-bodied species, while removal of small-bodied species had no effect on the density of large-bodied species, which implicates interspecific interference competition (Lemen and Freeman 1983). Experimental removals at ecotones indicated that competition between similar-sized species may be reduced by divergent habitat preferences (Schroder and Rosenzweig 1975).

Of the 31 extant heteromyid species and subspecies in California, 19 are listed as endangered, threatened, or species of special concern by the federal or state government (CNDDB 2017). Habitat loss and fragmentation are the most pervasive threats to heteromyids in California (Goldingay et al. 1997), and can result in smaller population sizes, lower migration rates and genetic connectivity, and increased extinction risk (Vandergast et al. 2007). Translocation—the intentional movement and release of organisms—is frequently used to mitigate the effects of development (i.e., habitat destruction) on heteromyids. Although many translocations of heteromyids have been conducted (Williams et al. 1993; O’Farrell 1999; Tennant et al. 2013; Shier et al. 2016; Tennant and Germano 2017; Saslaw and Cypher 2020), few have resulted in viable populations that persist over the long-term (Shier and Swaisgood 2012; Germano et al. 2013; Longland and Dimitri 2021; but see Shier et al. 2021). Understanding the mechanisms that reduce niche overlap and contribute to stable coexistence (e.g., a community of species that co-occur over long periods with members buffered from extinction, sensu HilleRisLambers et al. 2012) may lead to more effective conservation management (Seddon et al. 2007).

We tested for patterns of spatial and temporal niche partitioning and assessed species differences in resource selection of a rodent community through year-round trapping surveys and fine-scale habitat measurements. We were particularly interested in understanding how the smallest species in the community, the Los Angeles pocket mouse (*Perognathus longimembris brevinasus*), persists with its larger competitors. Pocket mice are behaviorally subordinate to the larger sympatric rodents (Chock et al. 2018) and do not appear to pilfer from the other species’ seed caches sufficiently to gain a competitive advantage (Chock et al. 2019). If niche partitioning in this granivore guild was driven by interference competition, we would expect the largest, dominant species (i.e., kangaroo rats, *Dipodomys* spp.) to have the largest influence on spatial and/or temporal species segregation. Alternatively, if niche partitioning was primarily caused by exploitative competition, we would expect greater spatial segregation between species in the same family than between species in different families, because diet overlap is greater within families than between families in this species assemblage (Table 1). These hypotheses are not mutually exclusive, however, as patterns of niche partitioning could be a product of both exploitative and interference competition.

**Table 1:**
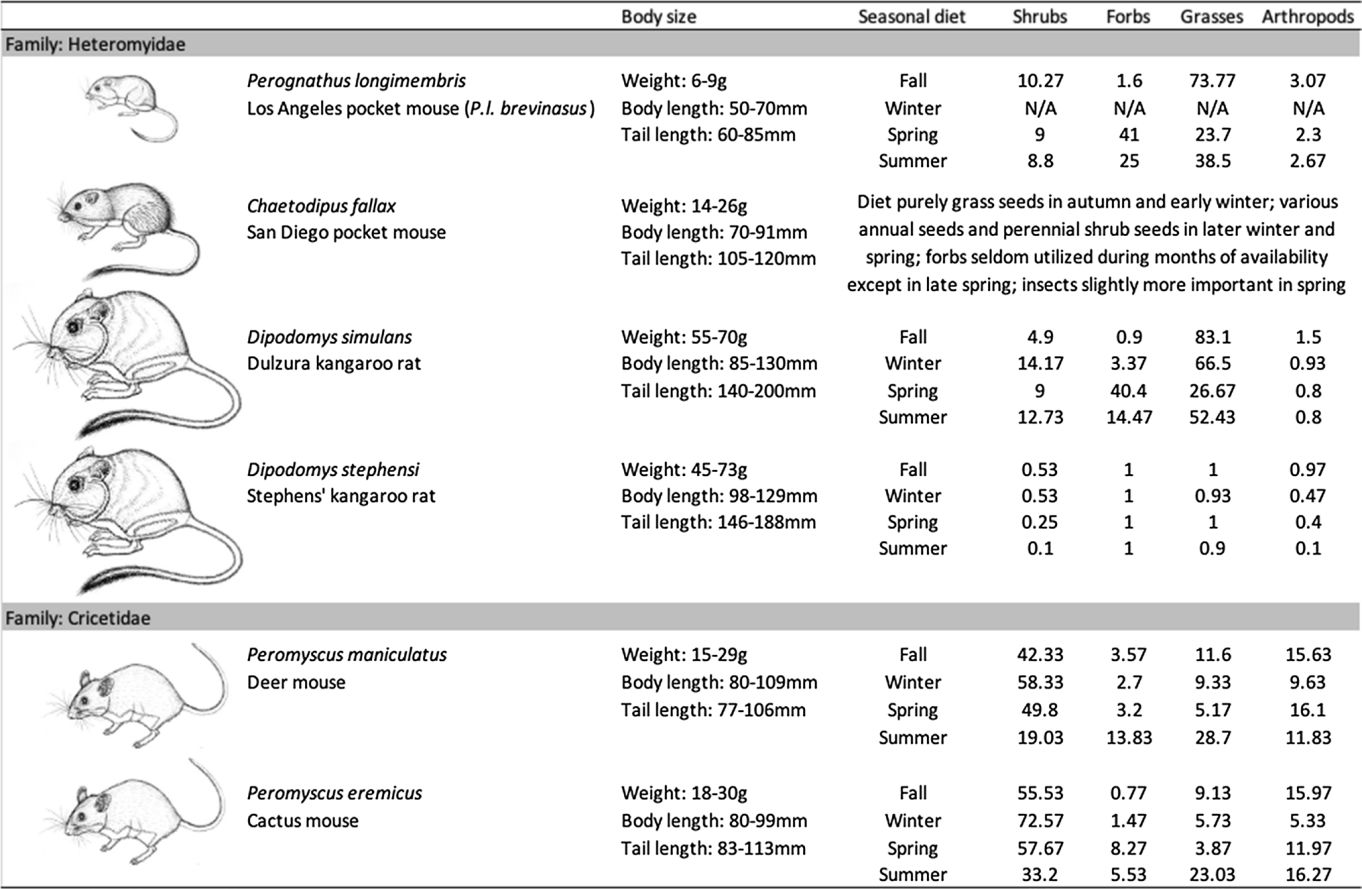
Descriptions of each species and their seasonal diet

Average body size measures taken from Reid (2006). Diet for all species, except Stephens’ kangaroo rat, is from Meserve (1976) and reported as an index of volume per fecal sample. Stephens’ kangaroo rat diet is from Lowe (1997) and reported as the frequency of occurrence in fecal samples

## Materials and methods

### Rodent community

**Fig. 1:**
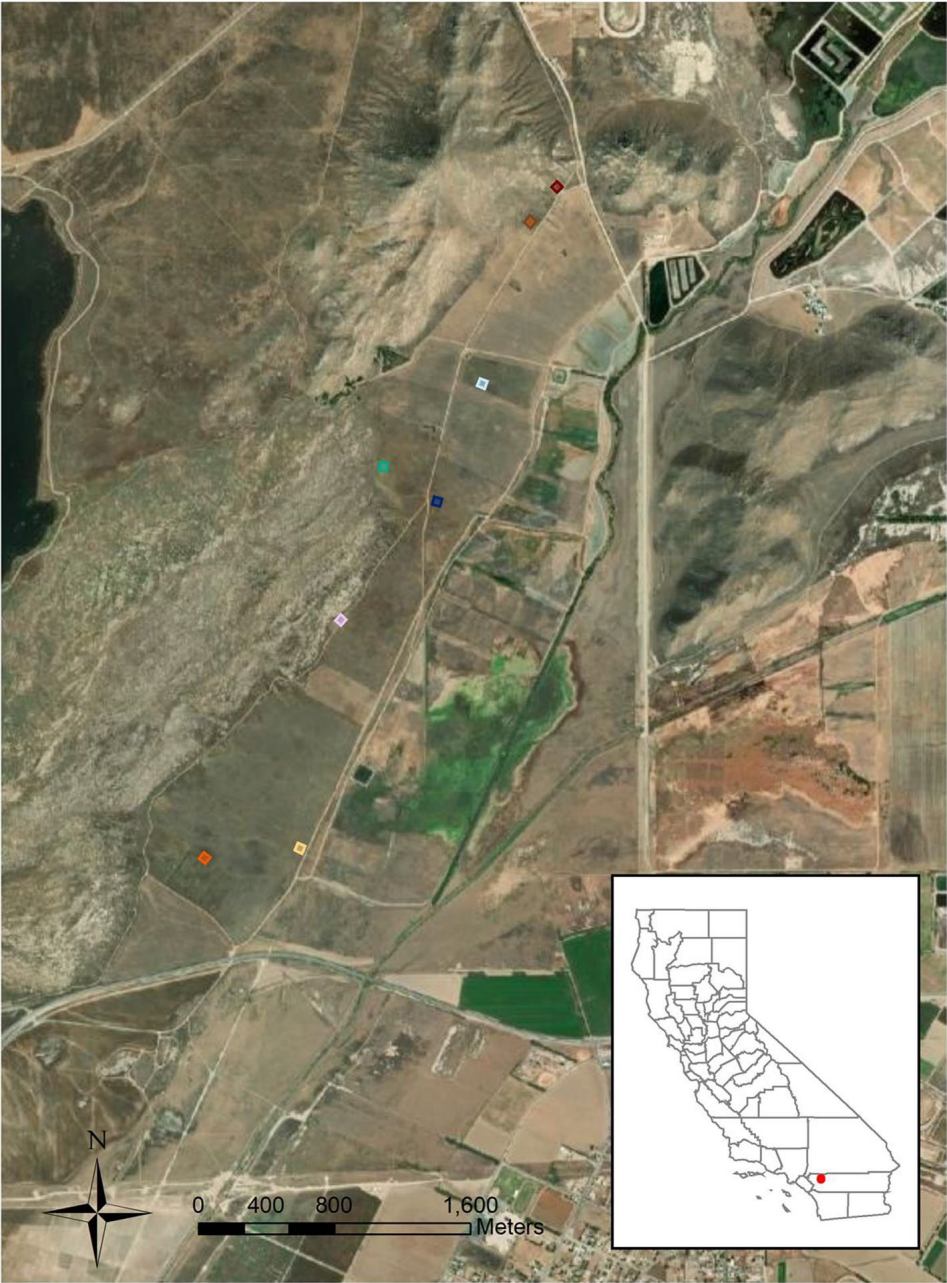
Aerial imagery of the San Jacinto Wildlife Area, California, USA. The locations of the trap grids used in this study are marked with colored squares. Each grid consisted of 49 traps (7 × 7 trap grid, 6.25 m spacing)

We conducted this research in the San Jacinto Wildlife Area, Riverside County, California, USA (33.13° N, 116.54° W) August 2015–July 2016 (Fig. 1). Six species of rodents occurred in sympatry at this site, including four heteromyid species at risk of extinction. The Los Angeles pocket mouse and San Diego pocket mouse (*Chaetodipus fallax*) are listed in the state of California as Species of Special Concern and the Dulzura kangaroo rat (*Dipodomys simulans*) is considered vulnerable. The Stephens’ kangaroo rat (*Dipodomys stephensi*) is listed as Threatened at the state level and Endangered at the federal level. Two common species from the family Cricetidae were also present: deer mice (*Peromyscus maniculatus*) and cactus mice (*Peromyscus eremicus*). These sympatric species have extensive dietary overlap (Table 1).

### Trapping

Eight trapping grids were established a minimum of 200 m apart to minimize the possibility of the same individuals being trapped on different grids (McNab 1963; Maza et al. 1973; Shier 2009) (Fig. 1). Sets of 49 traps were arranged in 7 × 7 grids with 6.25 m spacing. We used Sherman live-traps (Sherman Traps, Inc., Tallahassee, FL, USA) with modified shortened doors to avoid tail injury. To study these nocturnal species, we opened traps and baited with sterilized millet seed before dusk and checked three times during the night to quantify early (within 2 h of dusk), middle of the night, and late (within 2 h of dawn) activity. Animals were released at point of capture during each trap check. We closed traps during the late check. Each month we trapped 3 consecutive nights around the new moon to minimize variability in activity related to the lunar cycle (Prugh and Golden 2014) for a total of 14,112 trap nights and 42,336 trap-intervals.

All animals were individually tagged for identification. We used uniquely numbered ear tags for kangaroo rats, deer mice and cactus mice (Monel 1005-1, National Band and Tag Co., Newport, KY). For small-eared pocket mice we injected visible implant elastomer (Northwest Marine Technology, Inc., Shaw Island, WA, USA), which is visible under black light, in unique color combinations under the skin of the tail (Shier 2009). We recorded unique ID, sex, weight, reproductive condition, and trap location the first time we trapped each individual every month and ID and trap location on subsequent captures. This work was conducted under San Diego Zoo Wildlife Alliance IACUC protocol 15-002 and in compliance with State and Federal permits.

### Spatial activity patterns

To test whether species segregate or aggregate in space, we analyzed species co-occurrence patterns at trap locations on each of 8 survey grids over 4 seasons (Fall: August–October; Winter: November–January; Spring: February–April; Summer: May–July) using null model analyses. We constructed presence-absence matrices with species (*n* = 3–6) as rows and trap location (*n* = 49) as columns. A total of 32 matrices of grid-seasons (8 grids × 4 seasons) were assessed. All capture data, including re-captures, were included. We used the C-score (Stone and Roberts 1990) to measure the number of ‘checkerboard units’ of all species pairs in an assemblage. A checkerboard unit is calculated for species pair AB by *C*_AB_ = (*r*_A_ – *S*)(*r*_B_ – *S*) where *r*_A_ is the row total for species A, *r*_B_ is the row total for species B, and *S* is the total number of ‘sites’ (i.e., trap locations) that contain both A and B. Species that always occur together (complete spatial aggregation) will have a C-score of zero. The greater the segregation of species, the larger the C-score will be, to a maximum of *r*_A_*r*_B_ (complete spatial segregation). For each grid-season, pairwise C-scores were calculated for each species pair and then averaged over all possible pairs in the assemblage to obtain a community C-score.

To generate a null distribution, communities were randomized 5000 times using a fixed–fixed model (SIM9) in EcoSimR (Gotelli et al. 2015). The SIM9 algorithm preserves the number of occurrences of each species (row totals) and the number of species in each trap location (column totals). Species occurrences are random with respect to one another, which is an appropriate null model for detecting patterns of co-occurrence caused by species interactions (Gotelli 2000). If an observed C-score is small compared to the null distribution, this indicates that the species’ spatial activity patterns are aggregated. An observed C-score that is large compared to the null distribution indicates that the species’ spatial activity patterns are segregated (i.e., partitioned).

C-scores from different grid-seasons could not be directly compared because the species assemblages varied. Thus, to make these comparisons, we calculated the standard effect size (SES) of each grid-season, where SES is the number of standard deviations the observed community C-score is above or below the mean of the randomized assemblage (Gurevitch et al. 1992; Gotelli and McCabe 2002; Wittman et al. 2010). The SES is calculated as (*I*_obs_ – *I*_sim_)/*S*_sim_, where *I*_obs_ corresponds to the index for the observed assemblage, *I*_sim_ corresponds to the index for the null assemblages, and *S*_sim_ is the standard deviation of the null assemblages. SES values for each grid-season outside of the 95% confidence interval of the null SES distribution indicate either spatial segregation (positive values) or aggregation (negative values). We identified the species pairs that contributed most to the overall patterns of spatial activity as those with pairwise C-scores in the 95th percentile of all pairwise combinations in each gird-season community (Arrington et al. 2005; Pickles et al. 2012).

### Temporal activity patterns

We determined whether species segregate or aggregate diel (nightly) activity using null model analyses to examine the temporal overlap of species. Matrices with species (*n* = 3–6) as rows and time of night (*n* = 3 trap checks) as columns were constructed for each grid. Matrix entries were the total number of occurrences of each species during each sampling period (3 nights/month × 3 months within each season) in each grid. All capture data, including re-captures, were included. We used the Czekanowski index (Feinsinger et al. 1981) to quantify the area of intersection of two resource utilization (i.e., time of night) histograms for a pair of species. For species 1 and 2 the Czekanowski index is defined as

$$ O_{12} = O_{21} = 1 - 0.5\left( {\sum\limits_{i = 1}^{n} {\left| {p_{i1} - p_{i2} } \right|} } \right) $$

where *p*_*i*1_ is the proportion of occurrences of species 1 in a time interval (early, middle, or late) out of all times it was found during the sampling period. This symmetrical index ranges from 0 (no overlap; complete segregation) to 1 (complete overlap; aggregation). *O*_*ij*_ was calculated for each species pair then averaged over all species pairs in the assemblage for a given grid-season.

To generate the null distribution, communities were randomized 1000 times using randomization algorithm 3 (RA3) in EcoSimR (Gotelli et al. 2015) following methods by Albrecht and Gotelli (2001) and Wittman et al. (2010). RA3 retains the niche breadth (relative degree of specialization) of each species and randomly varies which resource categories (times of night) are used. Resource states were set as equiprobable, as time is assumed to be equally available to all species in the absence of species interactions. If an observed average *O*_*ij*_ is small compared to the null distribution, this indicates that the species’ nightly activity periods are segregated (i.e., partitioned). If an observed temporal overlap index is large compared to the null distribution, this indicates that the species’ nightly activity periods are aggregated.

Czekanowski indices from different grid-seasons could not be compared directly because they differed in the number of species present. We, therefore, compared standard effect size (SES) between grid-seasons, as described above for the spatial activity pattern analysis. SES values for each grid-season outside of the 95% confidence interval of the null SES distribution indicate either temporal aggregation (positive values) or segregation (negative values), and we again identified the species pairs that contributed most to the overall temporal activity patterns.

### Factors that predict niche partitioning

To determine which factors predict patterns of spatial and temporal partitioning, we ran generalized linear models in R 3.6.2 (R Core Team 2020) with C-score SES and Czekanowski SES as the dependent variables. The predictor variables were season, number of total captures in a grid-season, species richness (number of species), and the presence of each species in a grid-season. Deer mice were not included, as they were present in every grid-season.

### Resource selection

In addition to assessing the position of animals relative to one another (see Spatial Activity Patterns), we evaluated the resource selection of each species by comparing the microhabitat that was used relative to what was available (Johnson 1980; Boyce et al. 2002; Beyer et al. 2010). We restricted the evaluation of resource selection to the summer months (May–July 2016) when rodent activity was highest and annual herbaceous plants could be identified. Each trapping grid contained 49 non-overlapping 6 × 6 m pixels of habitat centered on the trap location. We considered an animal captured in a trap as using that pixel, and all 49 pixels as available to each individual on a grid as a way of systematically sampling availability (Benson 2013). With 27 sampling periods, we assume an individual had an opportunity to enter a trap in a pixel it used at least once. Traps were placed close together to provide multiple options within the expected range of each individual. Although traps were baited, an animal would need to be present in the pixel to detect the bait. While an animal could have visited pixels without entering the trap, our approach is similar to standard analysis of radiotelemetry data in which locations are taken at set intervals (Fieberg et al. 2010).

To quantify vegetation cover within each 6 × 6 m pixel in May and early June, we conducted visual surveys and estimated the percent cover of open ground, woody debris/leaf litter, forbs and grasses at ground level (< 10 cm height) and shrubs at crown height (Brehme et al. 2016). To measure soil texture, we collected soil samples at five locations on each grid. We scraped off the top layer of organic material and used a sharp hand trowel to cut down 20 cm and collected 100 g of soil. We used the Bouyoucos Hydrometer Method (Gee and Bauder 1986) to determine the percentage of sand, clay, and silt in each sample, then calculated composite estimates for each of the other 44 points on the grid (Supplemental Equation S1).

We used principal components analysis (PCA) to reduce the vegetation cover and soil variables to a smaller number of orthogonal axes. We compared features of the used versus available pixels for each individual using a binomial generalized linear mixed model (GLMM). We used a binomial (0/1) outcome variable, depending on whether the individual was caught at that trap location. All capture data, including re-captures, were included. The predictor variables (fixed effects) were the soil and habitat PC scores associated with each pixel. Individual ID and grid number were included as a random-effects. Models were fitted in R 3.6.2 with the ‘glmer’ function in the lme4 package (Bates et al. 2015).

## Results

In total, we captured of 757 individuals across the 6 study species: 112 Los Angeles pocket mice, 93 San Diego pocket mice, 51 Dulzura kangaroo rats, 135 Stephens’ kangaroo rats, 291 deer mice, and 75 cactus mice.

### Spatial activity patterns

**Fig. 2:**
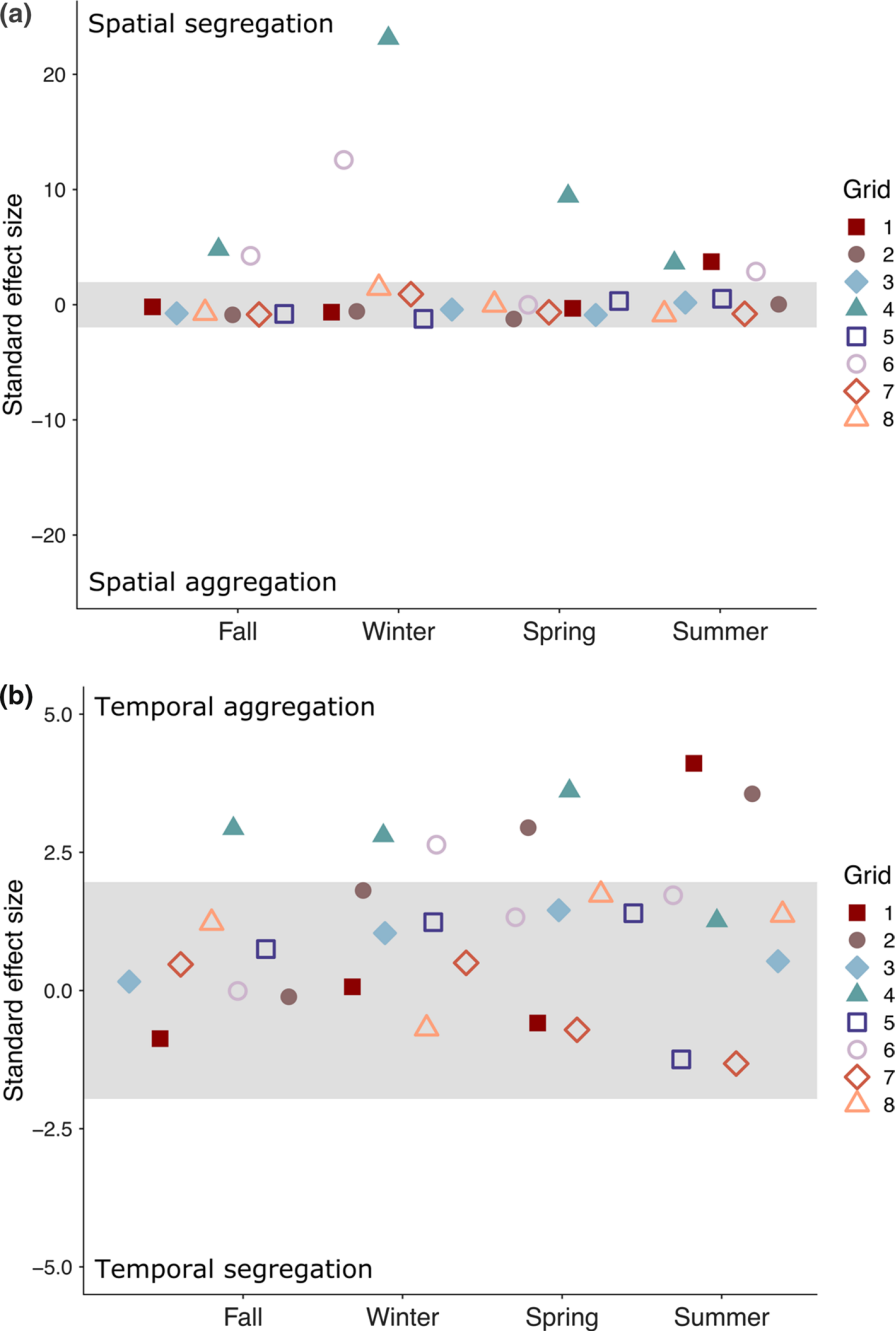
**a** Spatial co-occurrence of species in the rodent community. The plotted points are C-score standard effect sizes (SES) for each trapping grid and season. The shaded area depicts the 95% confidence interval of the null SES distribution. Points above the shaded area indicate spatial segregation while those in the shaded area do not deviate from the null expectation. Points below the shaded area would indicate spatial aggregation. **b** Temporal co-occurrence of species in the rodent community. The plotted points are Czekanowski Index SES for each trapping grid and season. The shaded area depicts the 95% confidence interval of the null SES distribution. Points above the shaded area indicate temporal aggregation while points in the shaded area do not deviate from the null expectation. Points below the shaded area would indicate temporal segregation. Please note the different scale in *y*-axes between (**a)** and (**b)**

**Fig. 3:**
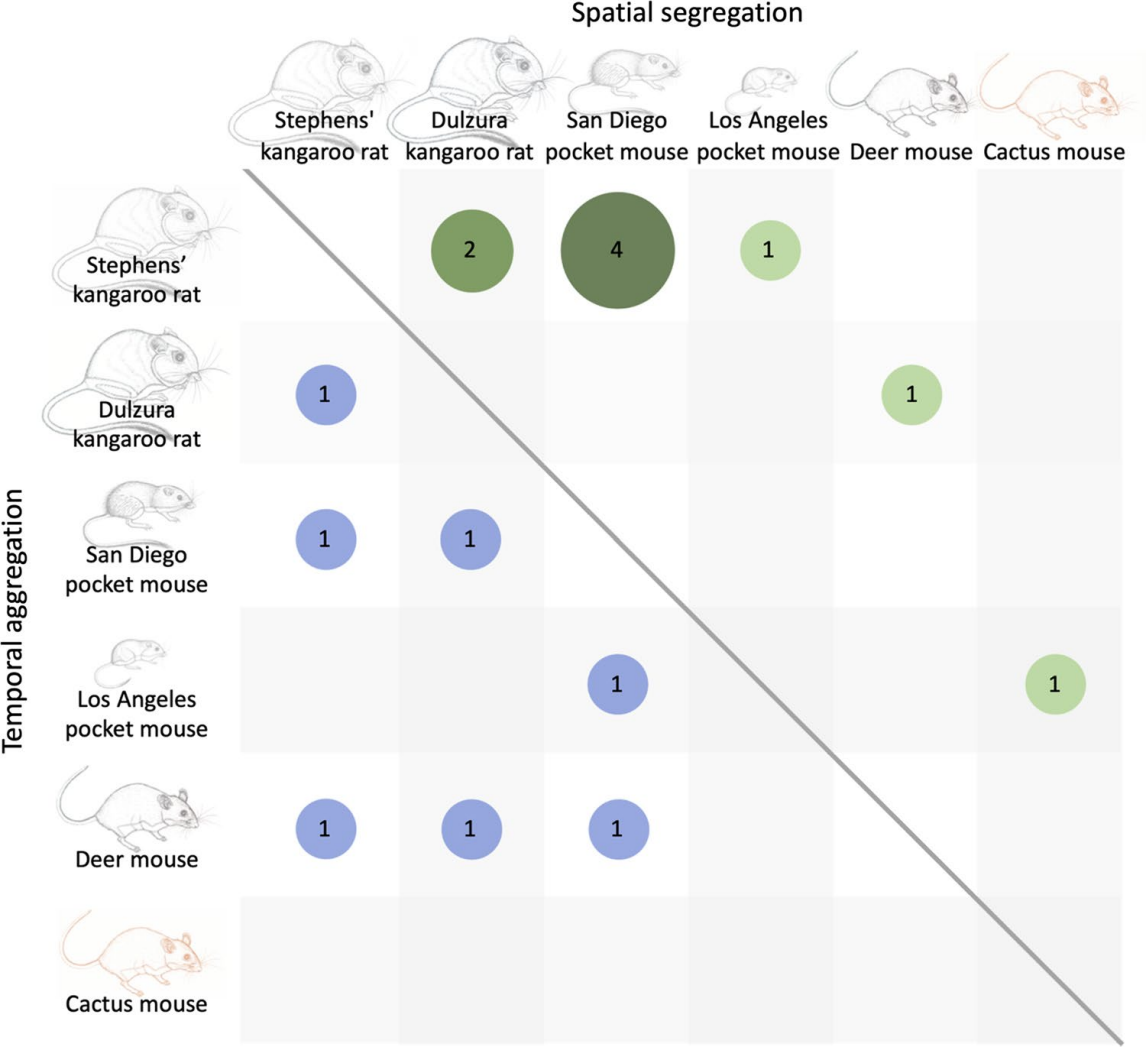
Species pairs contributing to overall community patterns of spatial partitioning and temporal aggregation. Values above the diagonal are the number of times a particular species pair had a pairwise spatial partitioning C-score in the 95th percentile of all pairwise combinations in each community (8 grid-seasons; 9 instances and 5 unique species pairs). Values below the diagonal are the number of times a particular species pair had a temporal aggregation Czekanowski Index in the 95th percentile (7 grid-seasons; 7 species pairs)

Spatial overlap was less than expected in 8 of 32 (25%) grid-season trapping bouts and never more than expected (Fig. 2a, Table S1). In the grid-seasons where spatial partitioning was detected, five species pairs made the greatest contribution to the overall C-scores (Fig. 3). Grids 4 and 6, which exhibited patterns of spatial segregation in multiple seasons (Fig. 2a) were the only two grids with both Stephens’ kangaroo rats and San Diego pocket mice present (Table S2), which was the species pair that most frequently contributed to patterns of spatial partitioning (Fig. 3). Consistent with the interference competition hypothesis, one or both kangaroo rat species was represented in all but one of the species pairs that contributed the most to the patterns of spatial partitioning (Fig. 3, Table S2). We found less evidence for spatial partitioning driven by exploitative (i.e., indirect) competition, which predicts greater spatial segregation between species of the same family than between species from different families. There were more cases of spatial partitioning within heteromyids (3 of 6 potential species pairs) than between heteromyids and cricetids (2 of 8 potential species pairs), but no partitioning between the two cricetids (Fig. 3).

### Temporal activity patterns

Temporal overlap was greater than expected in 7 of 32 (22%) grid-season trapping bouts, and never less than expected (Fig. 2b, Table S3). In the grid-seasons where temporal aggregation was detected, seven species pairs made the greatest contribution to the overall Czekanowski index (Fig. 3, Table S4). No one species or genus consistently drove the patterns of overlap in temporal activity, though kangaroo rats were included in 5 of the 7 species pairs (Fig. 3).

### Factors predicting niche partitioning

Over the course of the year, the only variables contributing significantly (and positively) to spatial niche partitioning among species were the presence of Stephens' kangaroo rats (model estimate 15.35 ± 4.93, *t* = 3.11, *p* < 0.01) and, to a lesser extent, Dulzura kangaroo rats (9.04 ± 4.14, *t* = 2.18, *p* = 0.04). No other variables included in the GLM—the presence of other species, season, total captures, or overall species richness—had detectable effects on the community C-score (Table S5). Temporal niche overlap, as measured by the temporal overlap Czekanowski index, was not predicted by any of the variables (Table S5).

### Resource selection

**Fig. 4:**
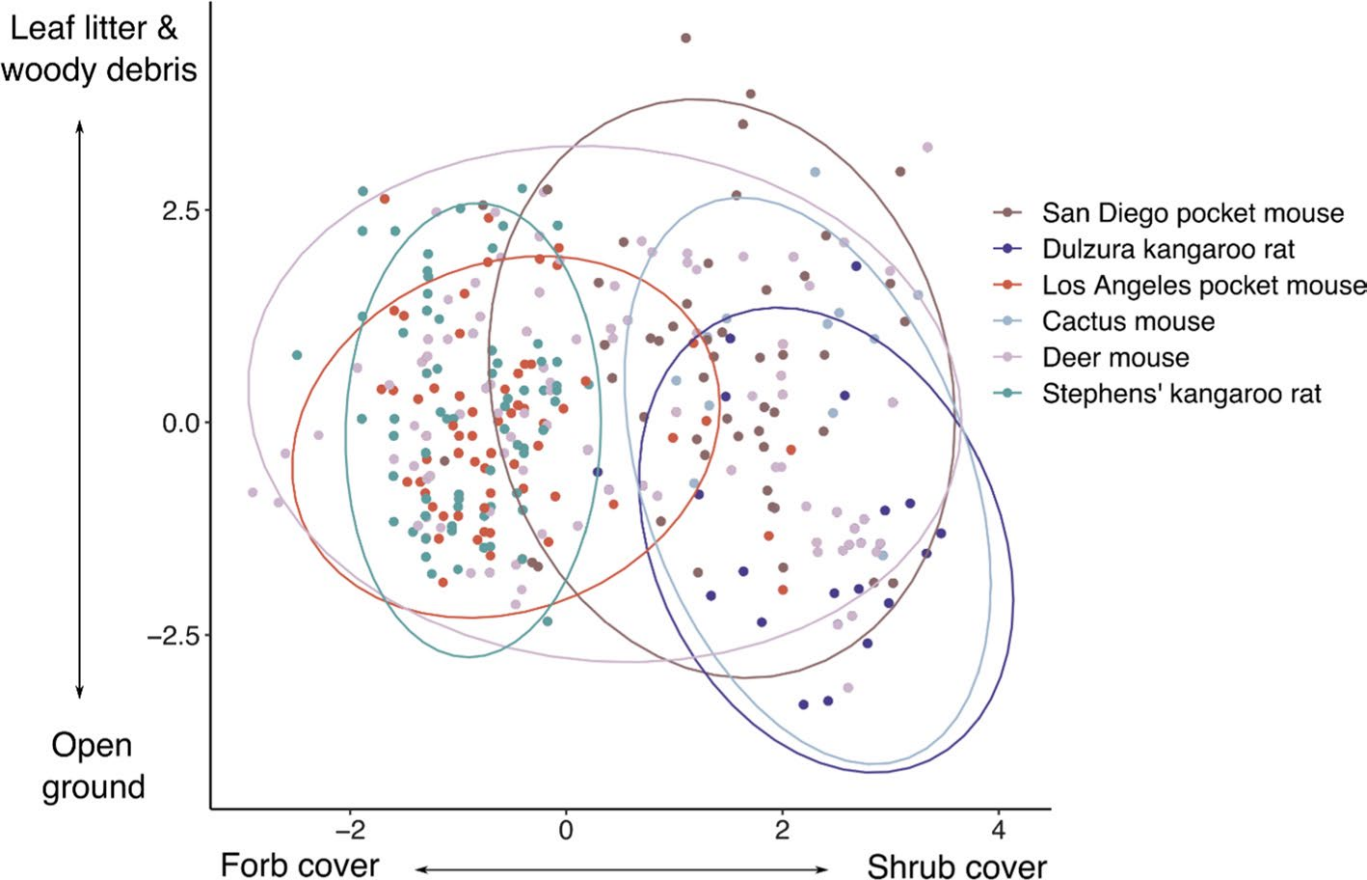
Species differences in habitat use as shown by principal components analysis of vegetation cover variables. Each point represents a trap location where the species was captured, and the values of PC1 (forb cover versus shrub cover) and PC2 (open ground versus leaf litter and woody debris) at that location. The ellipses enclose 95% of the points for a given species. Smaller ellipses indicate more restricted habitat use and non-overlapping ellipses represent species differences in habitat use

PCA of the vegetation cover variables revealed two axes of variation that together account for 75.0% of the variance (Table S6): shrub versus forb cover (PC1), and leaf litter and woody debris versus open ground (PC2). PCA of the soil texture variables revealed a single axis that accounts for 92.4% of the variance and represents the relative amounts of sand versus clay and silt (Table S6). The resource selection GLMM analyses revealed that most species in this rodent assemblage differ in their habitat preferences (Table 2). Los Angeles pocket mice used areas with more forb cover, leaf litter and woody debris. San Diego pocket mice and Dulzura kangaroo rats used areas with more shrub cover, open ground, and sandy soils, while Stephens’ kangaroo rats used areas with more forb cover and more open ground. Deer mice used areas with more woody debris and leaf litter, while cactus mice used areas with more shrub cover. The degree to which the species separated on the vegetation cover PCs is shown in Fig. 4.

**Table 2:** Resource selection GLMM models for each species in the rodent community

Species	Habitat axis	Estimate	SE	*t*	*P *value
Los Angeles pocket mouse	**Cover PC1**	**− 0.18**	**0.06**	**− 3.09**	**0.002**
	**Cover PC2**	**0.12**	**0.05**	**2.29**	**0.02**
	Soil PC1	0.03	0.06	0.53	0.59
San Diego pocket mouse	**Cover PC1**	**0.50**	**0.06**	**8.37**	**2e–16**
	**Cover PC2**	**− 0.17**	**0.06**	**− 2.90**	**0.004**
	**Soil PC1**	**− 0.23**	**0.07**	**− 3.29**	**0.001**
Dulzura kangaroo rat	**Cover PC1**	**0.52**	**0.10**	**5.25**	**1.54e–7**
	**Cover PC2**	**− 0.26**	**0.09**	**− 2.99**	**0.003**
	**Soil PC1**	**− 0.18**	**0.08**	**− 2.30**	**0.02**
Stephens’ kangaroo rat	**Cover PC1**	**− 0.43**	**0.07**	**− 6.21**	**5.43e–10**
	**Cover PC2**	**− 0.15**	**0.06**	**− 2.49**	**0.01**
	Soil PC1	0.01	0.08	0.06	0.95
Deer mouse	Cover PC1	0.00	0.04	− 0.10	0.92
	**Cover PC2**	**0.16**	**0.05**	**3.29**	**0.001**
	Soil PC1	0.05	0.05	0.97	0.33
Cactus mouse	**Cover PC1**	**0.65**	**0.10**	**6.45**	**1.14e–10**
	Cover PC2	− 0.18	0.10	− 1.81	0.07
	Soil PC1	0.06	0.073	0.87	0.39

Models also included random-effects terms for grid number and individual ID. Values in bold represent significant (*p* < 0.05) terms in each model

## Discussion

We sought to identify factors contributing to spatial and temporal partitioning, which could potentially reduce interspecific resource competition and stabilize coexistence, in a guild of six granivorous rodent species, four of which are at risk of extinction. We found evidence for spatial but not temporal partitioning. In fact, the diel activity of the species frequently overlapped more than expected by chance (Fig. 2b). Resource selection analysis, based on vegetation cover and soil texture, revealed differences in habitat use in nearly every species pair (Table 2). The extent to which these habitat associations correspond to the optimal habitat of each species or reflect the outcome of current or past interspecific competition remains to be determined. However, as predicted by the interference competition hypothesis, kangaroo rats—by far the largest and behaviorally dominant species in this granivorous rodent guild (Lemen and Freeman 1983; Chock et al. 2018)—strongly influenced the patterns of spatial segregation (Fig. 3 and Table S5). We found less support for the exploitative competition hypothesis, which predicted greater spatial segregation between species in the same family than between species in different families. Exploitative competition between heteromyids, but not cricetids, may have contributed to the observed patterns of spatial segregation.

In every grid-season where our analyses detected non-random temporal co-occurrence, the species were more aggregated than expected by chance (Fig. 2b). Patterns of temporal aggregation have also been found in a two-species rodent community in a cloud forest (Castro-Arellano and Lacher 2009), but another study with similar methods documented temporal segregation in a five-species rodent community in tropical semideciduous forest (Castro-Arellano and Lacher 2009). Temporal niche partitioning has a higher likelihood of facilitating coexistence as species richness increases (Kronfeld-Schor and Dayan 2003; Castro-Arellano and Lacher 2009), but is also expected to be more prevalent in habitats with low complexity (lack of multiple vertical strata) and low heterogeneity (little horizontal variation or patchiness) (August 1983; Vieira and Paise 2011). Although the community we studied had high species richness (6 species), it also had high habitat complexity and heterogeneity. Temporal partitioning may also be a viable mechanism for reducing competition if the limiting resource is renewed within the time separating the activity of species (Kronfeld-Schor and Dayan 2003), however, there is no evidence that this is the case for the seeds relied on by our study species. Two species of gerbil can coexist because the smaller species (*Gerbillus allenbyi*) forages more efficiently and is able to shift its foraging activity to later in the night, after the dominant species (*G. pyramidum*) has reduced seed densities to its higher giving-up density (Ziv et al. 1993). There is little evidence that small heteromyids forage more efficiently than large heteromyids (Reichman and Oberstein 1977; Chock et al. 2019), however, and thus shifting to foraging later in the night might not be a viable strategy for subordinate species, such as the Los Angeles pocket mouse.

Resource selection analysis revealed that the species pairs in our study that contributed the most to spatial niche partitioning also exhibited clear differences in habitat use. Stephens’ kangaroo rats occurred primarily in areas with high forb/low shrub cover and high bare ground/low leaf litter, and were segregated spatially from Dulzura kangaroo rats and San Diego pocket mice, both of which occupied areas with low forb/high shrub cover, low bare ground/high leaf litter, and sandy soils. Los Angeles pocket mice were found in areas with high forb/low shrub cover and low bare ground/high leaf litter, which most clearly distinguished them from Dulzura kangaroo rats and cactus mice (low forb/high shrub cover). The observed patterns of spatial segregation could be a product of differences in habitat preferences, with each species occupying the habitat type that yields the highest fitness returns (McLoughlin et al. 2006). But based on our finding that the presence of kangaroo rats increased spatial partitioning, it is likely that the observed patterns of habitat use are also influenced by competitive interactions, and that the presence of dominant species pushes subordinate species out of their preferred habitat, and thereby reduces their population densities.

Simulated territory intrusion tests in field enclosures, with a subset of the species in this study, showed that larger species dominated smaller species, and in particular, Los Angeles pocket mice were dominated by and avoided encounters with Dulzura kangaroo rats, regardless the individuals’ residency status (Chock et al. 2018). But testing the interference competition hypothesis at an appropriate scale and measuring population-level effects would require species removals or density manipulations. In other rodent communities, removal experiments have revealed strong evidence for competition, even among species with relatively low niche overlap. In a community similar to the one we studied, removal of the largest kangaroo rat species resulted in numerical increases in two smaller kangaroo rat species, and the removal of all three kangaroo rat species resulted in increases in density of four smaller granivorous species of rodents (including *Chaetodipus* and *Peromyscus*) (Brown and Munger 1985). In shortgrass prairie, when *Microtus ochrogaster* was removed from experimental plots, *Peromyscus maniculatus* increased in density on removal plots relative to control plots, providing evidence that *M. ochrogaster* affects the distribution and abundance of *P. maniculatus*, despite relatively small measured niche overlap (Abramsky et al. 1979). In montane cloud forest, a reciprocal removal experiment with singing mice (*Scotinomys* spp.) showed that the larger-bodied and behaviorally dominant species (*S. xerampelinus*) actively excludes its congener (*S. teguina*) from suitable habitat at higher elevations (Pasch et al. 2013).

Although ongoing interactions between species are most relevant from a conservation management standpoint, experimental removals might have little if any effect on the remaining species if differences between species in habitat use is the product of interspecific competition shaping habitat preferences in the evolutionary past (Connell 1980; Morris 2003), or divergence based on predator avoidance strategies (Brown et al. 1988). Some large-bodied bipedal species (e.g., kangaroo rats) are better able to detect and avoid predators in open microhabitat than small-bodied quadrupedal species (e.g., pocket mice), which could explain why the quadrupedal species are restricted to brush microhabitat (Brown et al. 1988; Kotler and Brown 1988). In the community we studied, however, two species of kangaroo rat that are very similar in body size nevertheless occupy different microhabitats (Stephens’ kangaroo rats are found in the open, and Dulzura kangaroo rats utilize areas with shrub cover). Thus, predator avoidance is unlikely to be the primary driver of coexistence in this community.

Successful translocations are essentially special cases of biological invasion, resulting in the re-establishment and persistence of a previously extirpated species in a resident community (Bright and Smithson 2001; Armstrong and Seddon 2008). A challenge facing conservationists is determining how to make target species more successful invaders to improve translocation outcomes. Just as community assembly theory provides a framework for understanding biological invasions (Pearson et al. 2018), it can play a role in how we assess a species’ translocation potential, particularly into an established resident community. Our results illustrate that understanding ecological differentiation and interference competition in a community assemblage can yield management recommendations to improve recovery of at-risk species.

The results of this study provide further justification for competitor exclusion in conservation translocations of heteromyid rodents (along with other common soft release techniques, such as predator exclusion, acclimation enclosures, and supplementary feeding (Batson et al. 2015)). However, they also suggest that such measures could be targeted at the most serious interference competitors. Both pocket mouse species in our study are probably impacted more by Dulzura and Stephens’ kangaroo rats than by each other or the two *Peromyscus* species. We caution that these results should not be assumed to apply to other heteromyid communities. For example, how pocket mice are affected by small-bodied kangaroo rats, such as the Endangered San Bernardino kangaroo rat (*Dipodomys merriami parvus*), remains to be studied.

Successful conservation interventions will create or restore the conditions that enable the at-risk species to establish and persist in an intact community with minimal (or, ideally, no) long-term intervention (e.g., Moseby et al. 2015). Direct removal of competing species may be necessary in the short-term to allow the target species to become established, particularly for conservation of small, subordinate species (Chock et al. 2018). However, permanently extirpating competing species is not a viable long-term strategy to facilitate persistence. Though unsurprising, our finding that nearly every rodent species in the community we studied differs from the others in habitat use suggests that habitat heterogeneity is a key part of what has enabled these species to coexist, and should be part of any conservation management plan involving these species. Simulating or restoring natural disturbance regimes (e.g., wild fire, episodic flooding) in protected areas could help forestall declines, or create suitable habitat for translocation of species that depend on the availability of early to mid-successional habitat, such as Los Angeles pocket mice, Pacific pocket mice (Miller et al. 2017) and San Bernardino kangaroo rats (Chock et al. 2020), while preservation or restoration of mature growth patches could help prevent declines of species that depend on climax vegetation, such as Dulzura kangaroo rats and San Diego pocket mice. Evidence of spatial segregation and unique habitat associations of different species suggest a mechanism through which habitat heterogeneity supports species diversity in this rodent guild.

Determining management actions that benefit both a target species and consider the habitat needs of sympatric species will be more effective for long-term conservation of guilds of similar species than single-species approaches. Community-level conservation is particularly relevant in regions such as southern California, where native habitats are already severely fragmented and diminished and multiple species in a community are threatened. We have framed this argument around specific species and limited data, but we hope that by providing such concrete examples of how fundamental ecological research can inform community-level conservation management that this idea might gain traction before it is too late to implement for many at-risk endemic species.

## Supplementary Information

Below is the link to the electronic supplementary material.

Supplementary file1 (PDF 294 KB)

## Data Availability

The data were deposited in figshare at 10.6084/m9.figshare.18295520.v1.

## Code availability

The code used during the study are available from the corresponding author on reasonable request.
