# Supplementary material for: Niche partitioning in an assemblage of granivorous rodents, and the challenge of community-level conservation
Source: Oecologia. 2022 Jan 15;198(2):553–65. doi: 10.1007/s00442-021-05104-5 (PMC8858926; doi:10.1007/s00442-021-05104-5)
Supplement: Supplementary file 1 — Supplementary file1 (PDF 294 KB) [file 442_2021_5104_MOESM1_ESM.pdf]

Electronic Supplementary Materials

Equation S1. We assessed the percent of sand, clay, and silt in five soil samples collected on each grid: the centrally located trap and the second trap in from each of the four corners. For each of the 44 trap locations (pixels) on a grid where we did not collect soil, we calculated the weighted distance from the center of each pixel (i) to each of the 5 soil sample points (j) using the equation

$$p_{ij} = \frac{(\frac{1}{d_{ij}})^2}{\sum_j (\frac{1}{d_{ij}})^2}$$

where $d_{ij} = \sqrt{[(x_i - x_j)^2 + (y_i - y_j)^2]}$ and x and y represent coordinates of each pixel. The weighted distance was then multiplied by the measured value for sand, clay, and silt for each censused point, resulting in a composite estimate for each of the remaining 44 points on the grid.

Table S1. Spatial activity pattern C-score results for all grid-seasons. Observed Index is the C-score value for the community present in each grid-season. Confidence intervals are the 95% two-tailed cut points for the null distribution generated from 5000 simulations. P-values indicate whether the observed index fell beyond the lower or upper confidence intervals. SES is the standard effect size, or the number of standard deviations the observed community C-score index is above or below the mean of the randomized distribution. Significant values are bolded.

| Grid | Season | Observed Index | Lower 95% CI | Upper 95% CI | Lower-tail P | Upper-tail P | SES |
|---|---|---|---|---|---|---|---|
| **1** | **Summer** | **93.70** | **82.30** | **90.80** | **0.99** | **0.00** | **3.73** |
| 1 | Spring | 92.20 | 89.60 | 97.80 | 0.44 | 0.58 | -0.31 |
| 1 | Winter | 57.50 | 54.00 | 63.00 | 0.20 | 0.84 | -0.65 |
| 1 | Fall | 65.50 | 61.90 | 71.60 | 0.48 | 0.54 | -0.19 |
| 2 | Summer | 77.80 | 74.93 | 81.74 | 0.56 | 0.46 | 0.03 |
| 2 | Spring | 82.83 | 82.50 | 87.67 | 0.10 | 0.95 | -1.24 |
| 2 | Winter | 48.33 | 46.50 | 57.17 | 0.31 | 0.70 | -0.58 |
| 2 | Fall | 96.00 | 95.10 | 100.10 | 0.21 | 0.82 | -0.88 |
| 3 | Summer | 43.00 | 40.33 | 51.00 | 0.73 | 0.39 | 0.18 |
| 3 | Spring | 64.67 | 64.00 | 76.00 | 0.38 | 0.89 | -0.89 |
| 3 | Winter | 19.67 | 19.67 | 29.67 | 0.70 | 1.00 | -0.42 |
| 3 | Fall | 46.33 | 46.33 | 51.00 | 0.39 | 1.00 | -0.75 |
| **4** | **Summer** | **125.00** | **115.40** | **122.40** | **1.00** | **0.00** | **3.62** |
| **4** | **Spring** | **95.10** | **77.20** | **83.60** | **1.00** | **0.00** | **9.42** |
| **4** | **Winter** | **171.67** | **130.67** | **136.83** | **1.00** | **0.00** | **23.13** |
| **4** | **Fall** | **120.10** | **106.10** | **114.50** | **1.00** | **0.00** | **4.81** |
| 5 | Summer | 66.00 | 60.00 | 73.33 | 0.83 | 0.43 | 0.51 |
| 5 | Spring | 110.67 | 106.00 | 117.33 | 0.77 | 0.31 | 0.32 |
| 5 | Winter | 121.00 | 121.00 | 135.00 | 0.10 | 1.00 | -1.23 |
| 5 | Fall | 67.83 | 66.83 | 75.00 | 0.16 | 0.84 | -0.80 |
| **6** | **Summer** | **129.10** | **121.80** | **128.20** | **0.99** | **0.01** | **2.88** |
| 6 | Spring | 103.83 | 100.50 | 109.67 | 0.58 | 0.46 | -0.01 |
| **6** | **Winter** | **142.00** | **122.67** | **128.00** | **1.00** | **0.00** | **12.57** |
| **6** | **Fall** | **80.07** | **74.13** | **78.20** | **1.00** | **0.00** | **4.24** |
| 7 | Summer | 108.00 | 106.67 | 118.67 | 0.20 | 0.89 | -0.79 |
| 7 | Spring | 60.33 | 59.00 | 68.00 | 0.48 | 0.81 | -0.66 |
| 7 | Winter | 41.33 | 39.00 | 44.67 | 0.83 | 0.33 | 0.91 |
| 7 | Fall | 30.83 | 30.50 | 36.00 | 0.35 | 0.74 | -0.85 |
| 8 | Summer | 44.33 | 44.00 | 50.67 | 0.36 | 0.80 | -0.84 |
| 8 | Spring | 37.33 | 37.33 | 37.33 | 1.00 | 1.00 | 0.00 |
| 8 | Winter | 66.00 | 60.67 | 69.00 | 0.93 | 0.10 | 1.45 |
| 8 | Fall | 18.00 | 18.00 | 23.33 | 0.57 | 1.00 | -0.72 |

Table S2. Spatial activity pattern C-score results for all species pairs in grid-seasons with overall community SES scores outside of the 95% confidence interval of the null SES distribution indicating either spatial segregation (positive values) or aggregation (negative values). The species pairs with pairwise C-scores in the 95$^{th}$ percentile (largest 5%) of all pairwise combinations in each gird-season contributed most to the overall patterns of spatial activity and are bolded.

| | Grid 4 Fall | Grid 6 Fall | Grid 4 Winter | Grid 6 Winter | Grid 4 Spring | Grid 1 Summer | Grid 4 Summer | Grid 6 Summer |
|---|---|---|---|---|---|---|---|---|
| Stephens' kangaroo rat - Dulzura kangaroo rat | 64 | N/A | **351** | N/A | **276** | N/A | 116 | N/A |
| Stephens' kangaroo rat - San Diego pocket mouse | 190 | **289** | 348 | **399** | 273 | N/A | **297** | **308** |
| Stephens' kangaroo rat - Los Angeles pocket mouse | 132 | **289** | N/A | N/A | 44 | N/A | 114 | 108 |
| Stephens' kangaroo rat - Deer mouse | 160 | 140 | 252 | 165 | 273 | N/A | 196 | 50 |
| Stephens' kangaroo rat - Cactus mouse | N/A | 220 | N/A | 189 | N/A | N/A | N/A | 144 |
| Dulzura kangaroo rat - San Diego pocket mouse | 28 | N/A | 10 | N/A | 4 | 55 | 10 | N/A |
| Dulzura kangaroo rat - Los Angeles pocket mouse | 77 | | | | 36 | 135 | 108 | |
| Dulzura kangaroo rat - Deer mouse | **217** | N/A | 39 | N/A | 0 | 70 | 0 | N/A |
| Dulzura kangaroo rat - Cactus mouse | N/A | N/A | N/A | N/A | N/A | 18 | N/A | N/A |
| San Diego pocket mouse - Los Angeles pocket mouse | 44 | 0 | N/A | N/A | 45 | 161 | 238 | 165 |
| San Diego pocket mouse - Deer mouse | 196 | 165 | 30 | 48 | 0 | 88 | 57 | 126 |
| San Diego pocket mouse - Cactus mouse | N/A | 13 | N/A | 15 | N/A | 42 | N/A | 33 |
| Los Angeles pocket mouse - Deer mouse | 93 | 165 | N/A | N/A | 0 | 92 | 114 | 60 |
| Los Angeles pocket mouse - Cactus mouse | N/A | 28 | N/A | N/A | N/A | **216** | N/A | 189 |
| Deer mouse - Cactus mouse | N/A | 128 | N/A | 36 | N/A | 60 | N/A | 108 |
| *95th percentile* | *207.55* | *289* | *350.25* | *346.5* | *274.65* | *194* | *270.45* | *254.45* |

Table S3. Temporal activity patterns by grid-season. Observed Index is the Czekanowski Index for the community present in each grid-season. Confidence intervals are the 95% two-tailed cut points for the null distribution generated from 1000 simulations. P-values indicate whether the observed index fell beyond the lower or upper confidence intervals. SES is the standard effect size, or the number of standard deviations the observed community C-score index is above or below the mean of the randomized distribution. Significant values are bolded.

| Grid | Season | Observed Index | Lower 95% CI | Upper 95% CI | Lower-tail P | Upper-tail P | SES |
|---|---|---|---|---|---|---|---|
| **1** | **Summer** | **0.96** | **0.89** | **0.94** | **1.00** | **0.00** | **4.11** |
| 1 | Spring | 0.87 | 0.86 | 0.91 | 0.34 | 0.67 | -0.59 |
| 1 | Winter | 0.93 | 0.92 | 0.95 | 0.60 | 0.41 | 0.07 |
| 1 | Fall | 0.83 | 0.82 | 0.87 | 0.24 | 0.77 | -0.87 |
| **2** | **Summer** | **0.92** | **0.86** | **0.91** | **1.00** | **0.00** | **3.56** |
| **2** | **Spring** | **0.83** | **0.77** | **0.82** | **0.99** | **0.02** | **2.95** |
| 2 | Winter | 0.95 | 0.93 | 0.96 | 0.94 | 0.07 | 1.81 |
| 2 | Fall | 0.91 | 0.90 | 0.93 | 0.54 | 0.47 | -0.11 |
| 3 | Summer | 0.84 | 0.79 | 0.89 | 0.75 | 0.28 | 0.53 |
| 3 | Spring | 0.95 | 0.91 | 0.96 | 0.95 | 0.11 | 1.45 |
| 3 | Winter | 0.94 | 0.86 | 0.94 | 1.00 | 0.33 | 1.04 |
| 3 | Fall | 0.91 | 0.90 | 0.92 | 0.45 | 0.61 | 0.16 |
| 4 | Summer | 0.87 | 0.83 | 0.89 | 0.89 | 0.12 | 1.26 |
| **4** | **Spring** | **0.90** | **0.81** | **0.88** | **1.00** | **0.00** | **3.61** |
| **4** | **Winter** | **0.84** | **0.78** | **0.83** | **0.98** | **0.02** | **2.80** |
| **4** | **Fall** | **0.91** | **0.84** | **0.91** | **0.99** | **0.01** | **2.93** |
| 5 | Summer | 0.62 | 0.62 | 0.70 | 0.35 | 1.00 | -1.25 |
| 5 | Spring | 0.92 | 0.72 | 0.92 | 1.00 | 0.06 | 1.40 |
| 5 | Winter | 0.93 | 0.88 | 0.94 | 0.90 | 0.14 | 1.24 |
| 5 | Fall | 0.63 | 0.60 | 0.66 | 0.82 | 0.22 | 0.75 |
| 6 | Summer | 0.92 | 0.88 | 0.94 | 0.93 | 0.07 | 1.72 |
| 6 | Spring | 0.70 | 0.61 | 0.73 | 0.89 | 0.12 | 1.33 |
| **6** | **Winter** | **0.92** | **0.87** | **0.92** | **0.99** | **0.01** | **2.64** |
| 6 | Fall | 0.82 | 0.80 | 0.85 | 0.61 | 0.39 | -0.01 |
| 7 | Summer | 0.88 | 0.88 | 0.95 | 0.10 | 0.00 | -1.32 |
| 7 | Spring | 0.77 | 0.76 | 0.85 | 0.28 | 0.83 | -0.71 |
| 7 | Winter | 0.52 | 0.45 | 0.56 | 0.67 | 0.44 | 0.50 |
| 7 | Fall | 0.58 | 0.51 | 0.65 | 0.75 | 0.27 | 0.47 |
| 8 | Summer | 0.79 | 0.75 | 0.87 | 0.90 | 0.13 | 1.37 |
| 8 | Spring | 0.57 | 0.48 | 0.57 | 1.00 | 0.06 | 1.73 |
| 8 | Winter | 0.84 | 0.84 | 0.89 | 0.68 | 1.00 | -0.69 |
| 8 | Fall | 0.78 | 0.71 | 0.80 | 0.90 | 0.16 | 1.22 |

Table S4. Temporal activity pattern results for all species pairs in grid-seasons with overall community SES scores outside of the 95% confidence interval of the null SES distribution indicating either temporal aggregation (positive values) or segregation (negative values). The species pairs with pairwise Czekanowski Index in the 95th percentile (largest 5%) of all pairwise combinations in each gird-season contributed most to the overall patterns of temporal activity and are bolded.

| | Grid 4 Fall | Grid 4 Winter | Grid 6 Winter | Grid 2 Spring | Grid 4 Spring | Grid 1 Summer | Grid 2 Summer |
|---|---|---|---|---|---|---|---|
| Stephens' kangaroo rat - Dulzura kangaroo rat | 0.872 | 0.941 | N/A | N/A | **0.966** | N/A | N/A |
| Stephens' kangaroo rat - San Diego pocket mouse | **0.986** | 0.92 | 0.948 | N/A | 0.93 | N/A | N/A |
| Stephens' kangaroo rat - Los Angeles pocket mouse | 0.924 | N/A | N/A | N/A | 0.885 | N/A | N/A |
| Stephens' kangaroo rat - Deer mouse | 0.962 | 0.933 | **0.952** | N/A | 0.931 | N/A | N/A |
| Stephens' kangaroo rat - Cactus mouse | N/A | 0.709 | 0.922 | N/A | N/A | N/A | N/A |
| Dulzura kangaroo rat - San Diego pocket mouse | 0.87 | 0.904 | N/A | 0.942 | 0.912 | 0.959 | **0.974** |
| Dulzura kangaroo rat - Los Angeles pocket mouse | 0.913 | N/A | N/A | N/A | 0.867 | 0.96 | 0.952 |
| Dulzura kangaroo rat - Deer mouse | 0.833 | **0.963** | N/A | 0.948 | 0.916 | 0.961 | 0.945 |
| Dulzura kangaroo rat - Cactus mouse | N/A | 0.726 | N/A | 0.733 | N/A | 0.964 | 0.882 |
| San Diego pocket mouse - Los Angeles pocket mouse | 0.91 | N/A | N/A | N/A | 0.923 | **0.992** | 0.957 |
| San Diego pocket mouse - Deer mouse | 0.961 | 0.867 | 0.909 | **0.977** | 0.868 | 0.966 | 0.945 |
| San Diego pocket mouse - Cactus mouse | N/A | 0.631 | 0.87 | 0.68 | N/A | 0.934 | 0.86 |
| Los Angeles pocket mouse - Deer mouse | 0.913 | N/A | N/A | N/A | 0.85 | 0.961 | 0.97 |
| Los Angeles pocket mouse - Cactus mouse | N/A | N/A | N/A | N/A | N/A | 0.941 | 0.841 |
| Deer mouse - Cactus mouse | N/A | 0.763 | 0.918 | 0.686 | N/A | 0.93 | 0.864 |
| *95th percentile* | *0.9752* | *0.9531* | *0.951* | *0.96975* | *0.95025* | *0.9803* | *0.9722* |

Table S5. Factors contributing to spatial segregation and temporal overlap in a Southern California rodent community**.** GLM results with standard effect size (SES) of spatial co-occurrence C-scores and temporal overlap Czekanowski indices as the dependent variables.

| Model | Model term | Estimate | SE | t | P-value |
|---|---|---|---|---|---|
| Spatial segregation | Season (Spring-Fall) | 0.52 | 1.89 | 0.27 | 0.99 |
| | Season (Summer-Fall) | -0.09 | 1.79 | -0.05 | 1 |
| | Season (Winter-Fall) | 1.49 | 2.31 | 0.65 | 0.92 |
| | Season (Summer-Spring) | -0.6 | 1.88 | -0.32 | 0.99 |
| | Season (Winter-Spring) | 0.98 | 2.08 | 0.47 | 0.97 |
| | Season (Winter-Summer) | 1.58 | 2.17 | 0.73 | 0.89 |
| | Total captures | 0.01 | 0.01 | 1.03 | 0.32 |
| | Richness | -0.11 | 1.8 | -0.06 | 0.95 |
| | **Stephens' kangaroo rat** | **15.35** | **4.93** | **3.11** | **0.01** |
| | **Dulzura kangaroo rat** | **9.04** | **4.14** | **2.18** | **0.04** |
| | Cactus mouse | 4.52 | 4.66 | 0.97 | 0.34 |
| | Los Angeles pocket mouse | -3.08 | 2.7 | -1.14 | 0.27 |
| | San Diego pocket mouse | 0.03 | 4.05 | 0.01 | 0.99 |
| Temporal overlap | Season (Spring-Fall) | 1.21 | 0.84 | 1.45 | 0.47 |
| | Season (Summer-Fall) | 0.63 | 0.79 | 0.8 | 0.85 |
| | Season (Winter-Fall) | 0.3 | 1.03 | 0.29 | 0.99 |
| | Season (Summer-Spring) | -0.58 | 0.84 | -0.69 | 0.9 |
| | Season (Winter-Spring) | -0.91 | 0.92 | -0.99 | 0.76 |
| | Season (Winter-Summer) | -0.33 | 0.96 | -0.34 | 0.99 |
| | Total captures | 0.002 | 0.003 | 0.8 | 0.43 |
| | Richness | 0.66 | 0.8 | 0.82 | 0.42 |
| | Stephens' kangaroo rat | 1.04 | 2.19 | 0.48 | 0.64 |
| | Dulzura kangaroo rat | 1 | 1.84 | 0.54 | 0.59 |
| | Cactus mouse | -0.46 | 2.07 | -0.22 | 0.83 |
| | Los Angeles pocket mouse | -1.26 | 1.2 | -1.05 | 0.31 |
| | San Diego pocket mouse | -0.58 | 1.8 | -0.32 | 0.75 |

Table S6. Vegetation cover and soil texture principal component loadings.

| Cover | PC1 | PC2 | Soil | PC1 |
|---|---|---|---|---|
| Shrubs | **0.63** | 0.03 | Sand | **-0.60** |
| Forbs | **-0.63** | -0.10 | Clay | **0.56** |
| Open ground | 0.24 | **-0.67** | Silt | **0.58** |
| Leaf litter and woody debris | 0.25 | **0.67** | | |
| Grass | -0.28 | 0.30 | | |
| Percent of variance | 40.67 | 34.28 | | 92.38 |
